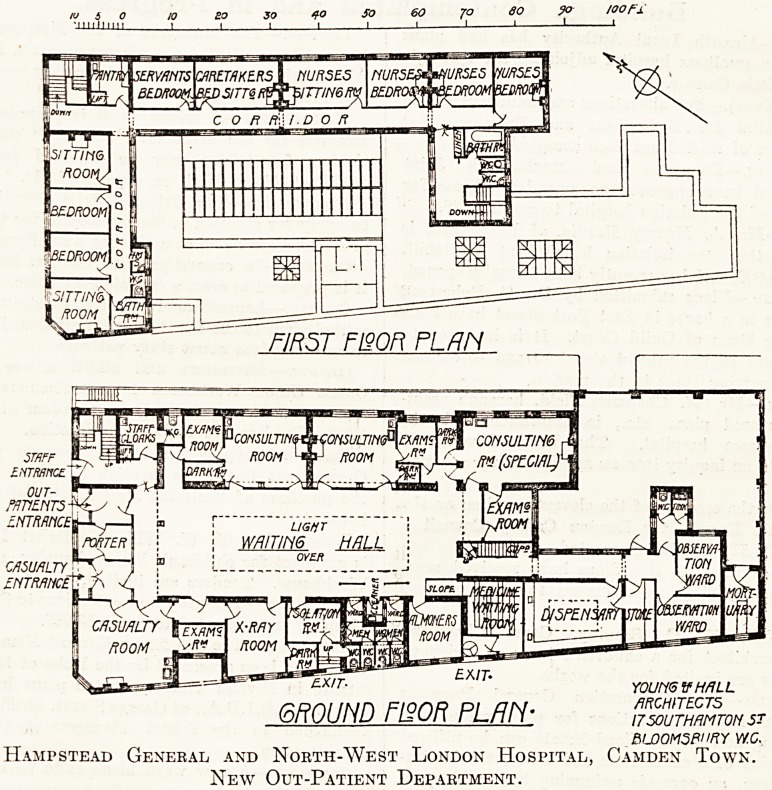# Hampstead General and North-West London Hospital New Out-Patient Department

**Published:** 1913-04-12

**Authors:** 


### HOSPITAL ARCHITECTURE AND CONSTRUCTION.

### Hampstead General and North-West London Hospital New Out-patient Department.

The new out-patient department of the Hampstead General and North-West London Hospital is situated at Camden Town, about two miles away from the hospital proper, which is at Haverstock Hill. The site is bounded on three sides by streets, to the north by Bayham Street, to the west by Hamilton Street, and to the south by Greenland Place. The portion facing Bayham Street is three storeys in height, containing basement, ground and first floors; that facing Hamilton Street is two storeys in height, having no basement; while the remainder, facing Greenland Place, is one storey only.

As will be seen from the accompanying plans, there are three entrances in Hamilton Street—one for the casualty department, one for the out-patients, and one for the staff; the porter's office, which is a glazed enclosure, controlling the entrances for casualties and out-patients, and also commanding a view of the waiting hall. Adjoining the casualty entrance is a large casualty room with an examination room attached, and a short passage connects the casualty room with the $x$-ray room, and also with the waiting hall. Attached to the $x$-ray room is a small dark room, next to which is the isolation room for infectious and doubtful cases, with a separate exit to the street.

The large top-lighted waiting hall is 65 feet long by 22 feet wide, and opening from it on the north-east side are two consulting rooms, each with separate examination and dark rooms, and an ophthalmic consulting room, also with a dark room and examination room attached. On the south-west side of the hall, and next to the isolation room, are the sanitary offices for patients, and a cleaners' cupboard, the almoner's room, the medicine waiting room, with direct exit and turnstile to Greenland Place, the dispensary and store room.

Hampstead General and North-West London Hospital, Camden Town.
New Out-Patient Department.

Beyond the dispensary is a special emergency department, comprising two observation wards with a sanitary annexe attached, for the reception of cases of a doubtful nature brought in at night, pending their removal, if necessary, to the hospital proper, and also for cases of patients brought in in a dying condition. Opening from the yard is a small mortuary.

On the upper floor are the quarters of the resident staff, comprising two sitting-rooms and two bedrooms for the medical officers; a sitting-room and three bedrooms for nurses, sanitary annexes, a pantry, servant's bedroom, and the caretaker's bed-sitting-room.

In the basement are the kitchen and offices, heating rooms, porter's sitting-room, stores, and a tradesmen's entrance.

The whole building is a most valuable and interesting example of a modern self-contained accident and emergency station and out-patient department—a type of institution which we hope to see become more general in the future, as it permits of the hospital proper for in-patients being situated in a suburban district and obtaining the benefits of a large site, whilst the accommodation for accidents and out-patients may be placed in the heart of the district for which it is required.

The building is heated throughout by means of a gas boiler and radiators, and the whole of the hot water for baths and sinks is obtained from a similar source. Gas is also laid on to all the fireplaces so that gas stoves can be installed if required.

The buildings have been designed and erected under the supervision of the architects, Messrs. Young and Hall, 17 Southampton Street, Bloomsbury, the contractors being Messrs. Ashby and Horner, of 8 Aldgate, London, E.

## Figures and Tables

**Figure f1:**